# Salmagundi

**Published:** 1886-02

**Authors:** 


### SALMAGUNDI.

EDITED BY E. G. BETTY, D.D.S.

THE "Dental Record" student still lives, having reached the XIIIth chapter.

JOHN B. GOUGH, the temperance lecturer, confessed the other night that his mouth had just been filled with false teeth.—*Clipping.*

NOVEMBER 11th, 1885, was particularly fatal to the dental profession in the loss by death of Drs. Riggs, Redman, and Bedford.

A CASE of death from cocaine applied to relieve the pain of a decayed tooth is reported by Prof. R. Ogden Doremus to the New York Medico-Legal Society.

PROF. HUXLEY has resigned the presidency of the Royal Society; a pension of $1,500 *per annum* has been conferred upon him in recognition of his scientific services.

JACK—"Grandma, have you good teeth?"

Grandma—"No, dear; unfortunately I have not."

Jack—"Then I'll give you my walnuts to keep till I come back."—*Boston Beacon.*

THE following extract from a private letter to the editor of Salmagundi, from Geneva, Switzerland, is significant: "The dental college started here a few years ago, is closed, a thing which has been a failure elsewhere; it may be possible some day, but that day has not yet come."

DR. C. G. ROEHR, of Milwaukee, Wis., writes to the *Medical Record* that he has used peroxide of hydrogen, with excellent results, as a disinfectant and stimulant for old ulcers and sinuses. When injected into sinuses containing exuberant granulations it destroys these, leaving uninjured the healthy tissues. There is very little pain, and a rapid improvement ensues.

---

Dr. Anton Kozura, of Buda-Pesth, says that aconite is an obtunder of sensitive dentine. He uses the alkaloid in a concentrated solution in ether, saturates a small piece of Japanese bibulous paper, lays it over the entire sensitive surface of the cavity and covers with gutta-percha. After the lapse of twenty-four hours or so, the cavity may be painlessly excavated, "provided there is no inter-current." It is essential for success that the interior of the cavity should be even, so that the remedy will penetrate to a uniform depth.

---

The following epitaph on the great English surgeon, John Hunter, was written by his wife:

"Here rests in awful silence, cold and still,
One whom no common sparks of genius fired,
Whose reach of thought Nature alone could fill,
Whose deep research to love of truth inspired,
Hunter! if years of toil and watchful care,
If the vast labors of a powerful mind,
To soothe the ills humanity must share,
Deserve the grateful plaudits of mankind,
Then, be each human weakness buried here,
Envy would rise to dim a name so bright.
Those specks which in the orb of day appear,
Take nothing from his warm and welcome light.

---

Reportorial.—The *Ohio State Journal*, the *Cincinnati Medical and Dental Journal*, and the Register, all had reporters at the Chillicothe meeting of the Ohio State Dental Society—Reorganized. Of the three, the Register only, had a complete and accurate report. The *State Journal* omitted the discussion on Pyorrhœa Alveolaris, on the ground that it was a "repitition" of another one, on the same subject, notwithstanding the fact that it was the first time Drs. Rehwinkel, Rawls, and Harlan had met upon the same floor to take part in the consideration of a most important and little understood affection.

The *C. M.* and *D. J.* committed a number of blunders, for one of which the *S. J.* takes it to task in the January number; the REGISTER is correct on the point mentioned by the *S. J.* It is well in this connection to remember that all those wishing to complete their files of transactions of the State Society can only do so by incorporating the reports of the REGISTER for the past two meetings.

